# Massachusetts Veterinary Association

**Published:** 1893-01

**Authors:** 


*Massachusetts Veterinary Association.*—At a meeting of the Massachusetts Veterinary Association, held at Boston on the evening of November 23d, 1892, the following resolutions were presented by a committee duly appointed at the previous meeting, October 26th, and adopted by vote of the members present.

(Signed) L. H. Howard, D.V.S., President.

(Signed) Austin Peters, M.R.C.V.S., Secretary.

*Whereas*, The members of the Massachusetts Veterinary Association regret that the Faculty of the recently established Veterinary School in Washington, while professing to have "arranged its curriculum to suit the requirements of modern Veterinary education," should have adopted a short two years' course of study, thereby lowering the standard of Veterinary education in this country, and ignoring entirely the unanimous opinion of the profession as expressed at the last annual meeting of the United States Veterinary Medical Association (Boston, September, 1892), and

*Whereas*, While we recognize the value of the laboratories of the Bureau of Animal Industry in the furtherance of Veterinary education, especially if connected with a long term or post-graduate school, it is the opinion of this Association that the establishment of the school as at present constituted is detrimental to the advancement of the Veterinary profession and a hindrance to the proper use of the laboratories of the Bureau for good work, be it therefore

*Resolved*, That this Association censures the action of the Veterinarians connected with this school who are in the employ of the United States;

That it is of the opinion that neither the best interests of the public nor the profession will be advanced by any such use of the staff or laboratories of the Bureau,

And that a copy of the above resolutions be forwarded to the Honorable the Secretary of Agriculture and others interested in the proper administration of the affairs of the Bureau of Animal Industry.

(Signed)

Committee on Resolutions: { Williamson Bryden, V.S.<br>J. F. Winchester, D.V.S.<br>John M. Parker, D.V.S.

